# Culture filtrates of lactic acid bacteria promote the growth and enhance the resistance of tomato seedlings to *Ralstonia solanacearum*

**DOI:** 10.1186/s12870-026-09213-2

**Published:** 2026-06-26

**Authors:** Salwa A. H. Gharib, Mona F. A. Dawood, Abdelrazek S. Abdelrhim

**Affiliations:** 1 Department of Agricultural Microbiology, Faculty of Agriculture, Minia University, El- Minia, 11432 Egypt; 2 Department of Plant Pathology, Faculty of Agriculture, Minia University, El-Minia, 11432 Egypt; 3 Botany and Microbiology Department, Faculty of Science, Assiut University, Assiut, 71516 Egypt

**Keywords:** Tomato wilt, Lactic acid bacteria, Secondary metabolism, Antioxidants, Biocontrol

## Abstract

*Ralstonia solanacearum* is a threatening pathogen that causes tomato wilt, which affects growing plants and reduces tomato yield. This study investigated the potential biocontrol of lactic acid bacteria cell-free culture supernatants (LAB-CFCS) against *R. solanacearum*. Four LAB, *B. longum*, *L. plantarum*, *L. salivarius*, and *L. rhamnosus* were examined as potential biological agents against *R. solanacearum.* The in vitro studies demonstrated the antibacterial activity of LAB-CFCS against *R. solanacearum* which suppressed bacterial growth and impeded biofilm formation. Organic acids (lactic and acetic) were detected in all LAB-CFCS. The highest lactic and acetic acid values (4.4 and 3.9 g/L) were observed in the CFCS of *L. plantarum* and *L. rhamnosus*, respectively. The application of LAB-CFCS on infected tomato plants reduced wilt incidence and severity. Treatment with CFCS of *L. salivarius* markedly reduced wilt incidence and severity in *R. solanacearum*–infected seedlings to 19.0% and 5.0%, respectively, compared with 100% and 80% in untreated infected controls. Infection by *R. solanacearum* elevates oxidative damage. Application of LAB-CFCS, particularly *L. rhamnosus* attenuates reactive oxygen species accumulation and lipid peroxidation while stimulating chlorophyll, carotenoids, antioxidant enzymes and salicylic acid-mediated defense signaling. In addition, LAB-CFCS significantly improved the contents of phenolics, flavonoids, terpenoids, alkaloids, and anthocyanins as a defense mechanism against *R. solanacearum* infection and modulated the activity of phenylalanine ammonia-lyase. All LAB-CFCS treatments showed promising in vitro and in vivo efficacy against *R. solanacearum*. The ability of LAB-CFCS to stimulate key physiological processes points to their dual role in promoting growth and mitigating biotic stress.

## Introduction

Tomato (*Solanum lycopersicum* L.) is the second most important vegetable crop globally, after potato, with about 100 million tons of fresh fruit being grown on 3.7 million hectares [1, 2]. Tomatoes are valued for their nutritional content and culinary versatility. However, tomato production is frequently constrained by a variety of pathogenic microorganisms, particularly fungi and bacteria, which cause significant yield losses and reduce fruit quality. Bacterial diseases pose serious challenges in tomato production. In this regard, *R. solanacearum* has proven to be a flexible bacteria capable of rapid adaptation to environmental changes and can weaken plants’ defense [3, 4].

Bacterial wilt caused by *Ralstonia solanacearum* is one of the most devastating plant diseases, affecting over 310 plant species across 54 botanical families, with tomato being a primary host [5]. *R. solanacearum* invades and colonizes tomato plants via root wounds; it eventually enters the xylem vessels leading to sudden wilting and causes the collapse of infected tomato plants [6–8].

Conventional management strategies of *R. solanacearum*, such as chemical bactericides a widely used strategy, have limited efficacy due to the emergence of resistant strains [9]. Biological control is an important management strategy that reduces the use of chemical agents against pathogens, which attenuate environmental pollution, ecosystem disruption, and residual chemicals on crops [10, 11]. Biological control using antagonistic microbes can reduce disease severity by producing antimicrobial metabolites and inducing systemic resistance [12]. The application of biocontrol agents has been used to suppress *R. solanacearum* [13, 14]. These agents employ diverse mechanisms against pathogens including antibiosis, competition for nutrients and niches, induced systemic resistance (ISR), and parasitism [15, 16].

Lactic acid bacteria (LAB) are promising candidates for the development of microbial biopesticides, as some strains hold Qualified Presumption of Safety (QPS) status from the European Food Safety Authority [10]. The application of LAB for controlling plant diseases and plant growth stimulation first appeared in the 1980s by Visser et al., [17] and Higa and Kinjo [18]. LAB strains have been reported as potential biological control agents against several bacterial phytopathogens [19–21]. LAB strains have the capacity to produce bioactive compounds, effective against a broad spectrum of bacterial and fungal phytopathogens and to enhance soil fertility, and promote plant growth [10, 22–25].

Among the various lactic acid bacteria (LAB), *Lactobacillus* and *Bifidobacterium* are considered two of the most important genera [26]. Both *Bifidobacterium bifidum* and *Lactobacillus fermentum* significant antifungal activity against *Aspergillus parasiticus* and demonstrated the ability to degrade the aflatoxins produced by this pathogen [10, 27]. *Lactobacillus* spp. have shown significant efficacy in suppressing a wide range of phytopathogens [20, 28, 29]. In particular, *Lactiplantibacillus plantarum* has studied as an effective biocontrol agent against several plant diseases, including fire blight in pear trees [19, 28]; bacterial soft rot in Chinese cabbage under field conditions [20], and *Ralstonia solanacearum* infection in tomato plants [30]. Moreover, combined applications of *Bacillus* and *Lactobacillus* spp. promoted the growth of beans and suppressed *Xanthomonas axonopodis* pv. *phaseoli* [31]. Beyond their agricultural significance, certain LAB strains also exhibit protective effects against mycotoxin-induced toxicity. For instance, *Lacticaseibacillus rhamnosus and Lactiplantibacillus plantarum* were shown to alleviate intestinal toxicity induced by deoxynivalenol (DON) [24, 32].

Due to the extensive and long-term application of LAB in food production systems, their physiological effects and the bioactive metabolites they produce have been thoroughly investigated. As a result, it has been classified as Generally Recognized as Safe (GRAS), with only minor exceptions [33]. This designation indicates that their application in edible crop production poses minimal risk to human health. Therefore, the present study aimed to: (i) evaluate the antagonistic activity of four LAB-CFCS, *Bifidobacterium longum*, *Lactiplantibacillus plantarum*, *Lactobacillus salivarius*, or *Lactobacillus rhamnosus*, against *R. solanacearum*; (ii) investigate the impacts of LAB-CFCS on wilt incidence and severity in *R. solanacearum*-infected tomato seedlings; and (iii) examine the biochemical responses of tomato plants infected by *R. solanacearum*-infected tomato seedlings when treated with various LAB-CFCS applications.

## Materials and methods

### Sources of pathogenic and lactic acid bacteria strains

Isolate of *R. solanacearum* previously isolated from potato plants collected from Assiut province Egypt and identified as race 3 (biovar II). The isolate was provided from Prof. Dr. Kamal Abo-El-Yousr, Department of Plant Pathology, Faculty of Agriculture, Assiut University, Assiut, Egypt.

The LAB strains were obtained from the Department of Agricultural Microbiology, Faculty of Agriculture, Minia University, Egypt. The LAB strains were isolated from fermented dairy products and molecularly identified as *Bifidobacterium longum* (AP014658.1), *Lactiplantibacillus plantarum* (MH544641.1), *Lactobacillus salivarius* (MG751346.1), *Lactobacillus rhamnosus* (AB889723.1) in a previous study by Ali et al., [34].

### Preparation of lactic acid bacteria cell-free culture supernatants (LAB-CFCS)

Lactic acid bacteria cell-free culture supernatants (LAB-CFCS) were prepared from the four LAB, *B. longum*, *L. plantarum*, *L. salivarius*, and *L. rhamnosus*, according to Pelyuntha et al., [35] and Yang et al., [36]. Flasks (250 mL) containing 100 mL of nutrient broth (NB) were inoculated with *B. longum*, *L. plantarum*, *L. salivarius*, and *L. rhamnosus* separately. The flasks were incubated at 37 °C while shaking overnight at 100 rpm. The bacterial cultures were transferred to 50 mL falcon tubes and centrifuged at 15,000×*g* for 15 min. The obtained supernatants were filtered using 0.22 μm filters (Guangzhou Jet Bio-Filtration Co., Ltd., Guangzhou, China). A loopful of the obtained CFCS was streaked on an NA plate and incubated for 24 h at 37 °C to confirm that no bacterial cells existed. After calibration, a pH meter (Pancellent Inc., China) was used for measuring the pH of CFCS obtained from the four bacterial strains. The pH values of *Bifidobacterium longum*, *Lactiplantibacillus plantarum*, *Lactobacillus salivarius*, and *Lactobacillus rhamnosus* were (4.2, 4.0, 3.9 and 4.1, respectively). The obtained LAB-CFCS were used for all the experiments presented in this work.

### Antibacterial activity of lactic acid bacteria cell-free culture supernatants (LAB-CFCS) against *R. solanacearum*

Agar diffusion technique described by Pompilio et al., [37] was used with minor modification to study the antibacterial activity of the selected LAB-CFCS, *B. longum*, *L. plantarum*, *L. salivarius*, and *L. rhamnosus* against *R. solanacearum.* Cell-free culture supernatants were prepared as described previously. 200 µL of *R. solanacearum* (10^8^ cells/mL) grown in nutrient broth (NB) for 24 h was added to the surface of solidified nutrient agar (NA) plates and spread using a sterile glass bacterial spreader. Six wells (5 mm diameter) were prepared on each plate. 10, 20, 30, 40 and 50 µL of each LAB-CFCS was loaded separately into the wells. A 50 µL of distilled water was added into control wells at the center of the plates. The plates were incubated at 28 °C for 48 h. The radius of the inhibition zones (the distance from the center of the well to the end of the inhibition zone) was used to represent the antimicrobial activity of LAB-CFCS. The experiment was implemented in a triplicate and repeated twice.

### Minimum inhibitory concentration (MIC) of lactic acid bacteria cell-free culture supernatants (LAB-CFCS)

The minimum inhibitory concentration (MIC) of the previously prepared culture filtrates of lactic acid bacteria (LAB-CFCS) was determined as described by Nguyen et al., [38]. LAB-CFCS were serially diluted using distilled water to different concentrations of 10, 20, 30, 40, 50 and 60%. *R. solanacearum* was grown on nutrient broth (NB) for 24 h. Bacterial growth was estimated by determining the optical density at 600 nm. Ten µL of the pathogenic bacteria *R. solanacearum* plus 50 µL of LAB-CFCS (*B. longum*, *L. plantarum*, *L. salivarius*, and *L. rhamnosus*) were added separately into different wells of a 96-well microtiter plate. The final volume of each well was adjusted to 200 µL considering the concentrations of tested LAB-CFCS. The plates were incubated for 24 h at 28 °C. The growth of *R. solanacearum* was measured at optical density of 600 nm (OD_600_) d after the incubation period using a microplate photometer (Thermo Fisher Scientific Inc., Waltham, MA, USA). Each concentration was represented by three replicates. The assay was repeated twice to confirm the obtained results.

### Inhibition of *R. solanacearum* biofilm formation

The inhibitory effect of LAB-CFCS on biofilm formation by *R. solanacearum* was tested according to O’Toole et al., [39] with minor modifications. Fifty µL of 24 h old *R. solanacearum* (10^8^ cells/mL) culture was mixed with 950 µL NB media and placed into a 1.5 mL Eppendorf tube. *R. solanacearum-*inoculated tubes were incubated at 30 °C for 24 h. A volume of 500 µL of 40% LAB-CFCS of each lactic acid bacteria was added to each tube separately. A 500 µL of NA media was added to the control tube, and then were incubated at 30 °C for 24 h. After incubation, the culture media was discarded to get rid of free *R. solanacearum* cells. The tubes were gently rinsed three times with sterile distilled water. The tubes were left to dry for 30 min at 60 °C to fix the bacterial membrane. The biofilm of pathogenic bacteria was stained using 1% crystal violet (CV). The stained biofilm was left at room temperature for 30 min. 200 mL of 95% ethanol was added to each tube to solubilize the crystal violet dye bound to the pathogenic bacteria biofilm. The OD of each tube was measured using a spectrophotometer (T80 UV/VIS, PG Instruments Ltd, England) at 530 nm. The experiments were implemented in a triplicate and performed twice to confirm the obtained results.

### In vivo effect of lactic acid bacteria cell-free culture supernatants (LAB-CFCS) on tomato infected with *R. solanacearum*

The cell free culture filtrates efficacy of four LAB-CFCS, *B. longum*, *L. plantarum*, *L. salivarius*, and *L. rhamnosus* was investigated against *R. solanacearum*-infected tomato following the method described by Sun et al., [40] with minor modifications. Seeds of tomato plants (variety 054 F1, obtained from Gaara Seeds Co., Egypt) were placed in a Petri dish containing wet filter paper. The dishes were incubated in a growth chamber at 27 ± 2 °C and 60% relative humidity and left to germinate for one week. The germinated seeds were transferred to 209-hole trays containing sterilized mixed soil clay and peat moss (SAB TORF 400, SAB Syker Agrarberatungs. Und Handels Gmbh & CO., Germany) at a 1:1 weight ratio. At the four leaf-stage, seedlings were carefully uprooted from the soil and were gently washed using tap water. The tips of the roots were cut using sterilized scissors and dipped in *R. solanacearum* suspension adjusted at 10^7^ cells/mL. The bacterial suspension was prepared from *R. solanacearum* grown on NA for 24 h at 30 °C. The control seedlings were dipped in water instead of the bacterial suspension. The seedlings were transplanted into 3-inch pots with one seedling/pot. Each 10 pot represents one replicate, and each treatment was represented by five replicates with total of 50 plants per treatment. After planting, the pots were transferred to the greenhouse, and each pot was drenched with 30 mL of the four LAB-CFCS separately. The pots were drenched with LAB-CFCS 5 and 10 days after transplanting. The control pots received water instead of LAB-CFCS. The experiment was designed in a complete randomized design (CRD) and repeated twice. Two weeks after inoculation, the seedlings were uprooted, length as well as fresh and dry weight of seedlings’ roots and shoots were measured. Also, wilt incidence and severity were estimated for each treatment using the following equations:

$$\text{Wilt incidence}\ (\mathrm{DI}\%) = (\text{No. of wilted plants/No. of total plants}) \times 100$$

Wilt severity was assessed according to Chiang et al., [41] using a wilt rating scale (0 to 5), where 0 = healthy seedling with no wilt symptoms, 1 = one wilted leaf (20% wilted tissues), 2 = two wilted leaves (40% wilted tissues), 3 = three wilted leaves (60% wilted tissues), 4 = wilting of all leaves without an apex (80% wilted tissues), and 5 = wilting of the entire plant (100% wilted tissues).

Disease severity (DS) = [(Ʃ (rating scale × no. of plants in the rating scale)/ (Total no. of plants × highest rating scale)] × 100.

### Quantification of lactic and acetic acids produced by lactic acid bacteria

Quantification of lactic and acetic acids produced by *B. longum*, *L. plantarum*, *L. salivarius*, and *L. rhamnosus* was conducted according to Scalabrini et al., [42]. Cell-free culture supernatants (CFCS) of the tested bacteria were prepared as described previously. A 30 mL of each supernatant was transferred into a 50 mL falcon tube. The tubes were centrifuged for 10 min/10,000×*g*. The obtained supernatant was analyzed using high-performance liquid chromatography (HPLC; Bio-Rad, Hercules, CA, USA) equipped with an Aminex HPX-87 H column (300 × 7.8 mm); the mobile phase was a 5 mM H_2_SO_4_ solution at a flow rate of 0.3 mL/min and a temperature of 70 °C, with UV detection at 210 nm. Each individual acid standard was prepared and chromatographed separately to determine its retention time. Calibration curves for each acid were generated using five concentration levels of standard mixtures, with three injections at each level.

### Effect of lactic acid bacteria cell-free culture supernatants (LAB-CFCS) on tomato seedlings physiological and biochemical traits under *R. solanacearum* infection

Using the same treatments and experimental design of the greenhouse experiment, fresh tomato leaves harvested from the middle branches of 4 weeks old tomato plants, were used for measuring the physiological and biochemical traits.

### Pigment content

Chlorophyll a, b and carotenoids were measured in fresh tomato leaves (0.05 g) suspended in 5 mL of ethyl alcohol (95%) using equations recommended by Lichtenthaler [43].

### Organic acids

The content of organic acids was estimated by acid–base titration as described by Zhang et al., [44]. Fresh samples were mixed with distilled water, centrifuged, the supernatant was extracted, diluted to 50 mL by distilled water; then 25 mL of diluted extract was mixed with 100 µL phenolphthalein and titrated with NaOH to reddish (for 30 s, the color did not fade), and the amount of NaOH was estimated to calculate the level of organic acids.

### Secondary metabolites

#### Flavonoids and anthocyanins content

Anthocyanins were quantified based on the method of Krizek et al., [45]. Total flavonoid content was colorimetrically assessed as described by Zou et al., [46] using aluminum chloride with slight modifications. Briefly, 100 mg of leaf tissue was ground to a fine powder using liquid nitrogen, extracted with 1.0 mL of methanol, then centrifuged at 10,000× *g* for 10 min. Subsequently, about 0.5 mL of the supernatant was mixed with 2 mL of distilled water and 150 µL of 5% sodium nitrate and incubated for 6 min. After incubation, 150 µL of aluminum chloride (10%) and 2 mL of sodium hydroxide (1 M) were added and re-incubated at room temperature for 15 min. The absorbance of the mixtures was measured at 510 nm.

#### Phenolic compounds

Phenolic compounds were determined using a Folin–Ciocalteu-based method as described by Kofalvi and Nassuth [47] using gallic acid as a standard curve. Total phenolics were expressed as mg g^− 1^ FW. Briefly, 300 mg of fresh leaf tissue was extracted in methanol (50%) in a water bath (70 °C) for one hour. The methanolic extract was mixed with distilled water + Folin–Ciocalteu’s reagent + Na_2_CO_3_ at room temperature. After 20 min, the absorbance spectrum was measured at 725 nm.

#### Total alkaloids

Total alkaloid content of tomato leaves was measured using the 1,10-phenanthroline method, as described by Singh et al., [48]. The reaction mixture contained the ethanolic extract, FeCl_3_, and 1,10-phenanthroline in ethanol and was incubated at 70 °C. The absorbance was read at 510 nm, and the total alkaloid content was calculated from the calibration curve of colchicine and expressed as micrograms per gram FW.

#### Total tannins

Total tannins in plant leaves were estimated using the Folin-Ciocalteu reagent, described by Polshettiwar et al., [49]. The leaves extract with distilled water was mixed with Folin-Ciocalteu reagent, followed by sodium carbonate solution. The absorbance was measured against prepared reagent blank at 775 nm using a spectrophotometer. Total tannins in the samples were expressed as mg of tannic acid equivalent g^–1^ dry weight. All samples were analyzed in triplicate.

#### Total terpenoids

The level of total terpenoids was carried out by a colorimetric method described by Fan and He [50]. Fresh leaves were dissolved in 1 mL of dichloromethane, then the extract was mixed with vanillin dissolved in glacial acetic acid and heated at 60 *◦*C for 45 min, followed by cooling in ice-water. Terpenoid content at 548 nm was expressed as milligrams ursolic acid equivalents (mg ursolic acid/g extract).

#### Oxidizing agents

Reactive oxygen species (ROS) were assessed by determining superoxide anions O_2_^─●^ (µg g^− 1^ FW) and hydrogen peroxide (µmol g^− 1^ FW, H_2_O_2_) via the published methods of Yang et al. [51] and Mukherjee and Choudhuri [52], respectively.

Lipid peroxidation was detected as described by Madhava Rao and Sresty [53] with some modifications. The thiobarbituric acid reaction was used to determine lipid peroxidation in tomato leaves by monitoring malondialdehyde formation.

### Signaling molecules

#### Salicylic acid (SA)

Fresh tomato leaves were used for the determination of SA as described by Warrier et al., [54]. Briefly, 100 mg of leaf tissue was ground to a fine powder using liquid nitrogen and extracted with 1.0 mL of extraction solvent (chloroform, amyl alcohol, ether, and ethanol), then centrifuged at 10,000× *g* for 10 min. subsequently, 100 µL of the supernatant with up to 3 mL of freshly prepared ferric chloride (0.1%) until the development of a violet color. The absorbance of the complex was measured using spectrophotometry at 540 nm.

#### Hydrogen sulfide (H_2_S)

The hydrogen sulfide (H_2_S) content in tomato leaves (liquid nitrogen and frozen leaves at − 80 °C) was estimated based on the method of Nashef et al. [55].

#### Nitric oxide content (NO)

Nitric oxide (NO) content was indirectly determined as described by Ding et al. [56] and modified by Hu et al. [57]. Briefly, NO was reacted with Greiss reagent (l% sulfanilamide and 0.1% N-(l-naphthyl)-ethylenediamine dihydrochloride in 5% phosphoric acid) was added for 30 min at room temperature till the appearance of azo-dye violet color, which was measured spectrophotometrically at 550 nm. NaNO_2_ was used to prepare the standard curve, and the NO content was expressed as nmol g^− 1^ FW.

#### Antioxidant enzymes

The antioxidative enzymes in tomato leaves were monitored by screening the specific activities of catalase (CAT, U mg^− 1^ protein g^− 1^ FW min^− 1^), superoxide dismutase (SOD, U mg^− 1^ protein g^− 1^ FW min^− 1^), ascorbate peroxidase (APX, µmol mg^− 1^ protein g^− 1^ FW min^− 1^), guaiacol peroxidase (POD, (U mg^− 1^ protein min^− 1^) using the recommended procedures of Noctor et al., [58], Misra and Fridovich [59], Silva et al., [60] and Zaharieva et al., [61], respectively.

Phenylalanine ammonia-lyase (PAL; EC 4.3.1.5) and polyphenol oxidase (PPO; EC 1.10.3.1) activities were examined using the protocol of Havir and Hanson (2002) [62] and Kumar and Khan [63], respectively.

### Statistical analysis

The laboratory and greenhouse experiments were performed using a completely randomized design (CRD). Each experiment was conducted at least twice, with five replicates per treatment. Only data from one trial were presented, as the values of each pair of repeated trials were similar. Analysis of variance (ANOVA-one way) was used to test the significance difference in laboratory and greenhouse experiments. A Tukey’s honestly significant difference (HSD) test was used for post-hoc analysis at *p* ≤ 0.05. The data were analyzed using JMP data analysis software version 14.

## Results

### Antibacterial activity of lactic acid bacteria cell-free culture supernatants (LAB-CFCS) against *R. solanacearum*

**Fig. 1:**
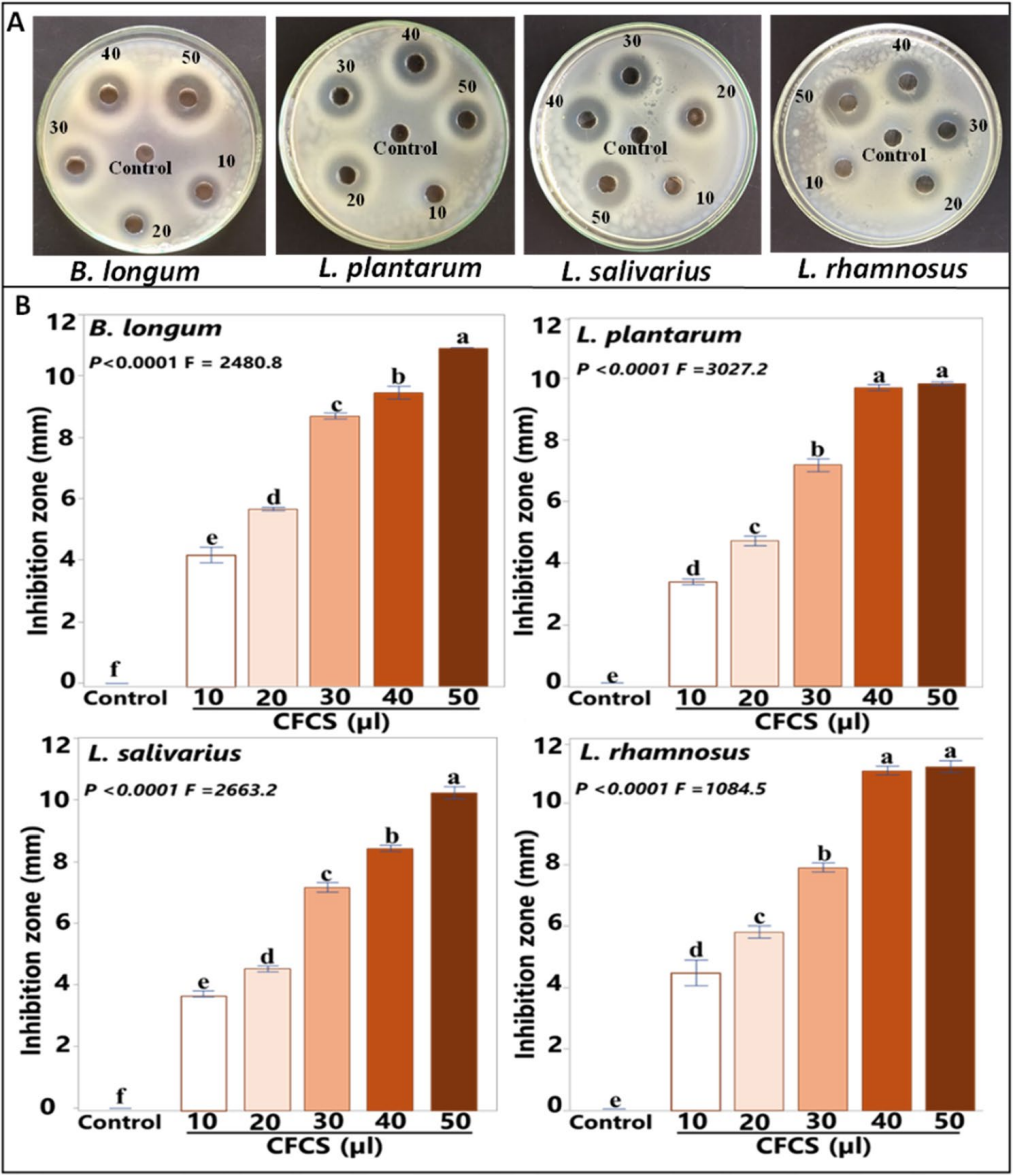
Antibacterial activity of lactic acid bacteria cell-free culture supernatants (LAB-CFCS) against *R. solanacearum*. **A** Agar diffusion technique, each Petri dish contains six wells loaded with 10, 20, 30, 40 and 50 µL CFCS of four lactic acid bacteria, namely *B. longum*, *L. plantarum*, *L. salivarius*, and *L. rhamnosus*. **B** The inhibition zones of *R. solanacearum* resulted from the LAB- CFCS. The data presented as means ± standard deviation of five biological replicates. Different letters indicate statistically significant differences among treatments, while the same letters indicate no significant differences between them according to Tukey’s honestly significant difference test (*p* < 0.05)

Cell-free culture supernatants of the four lactic acid bacteria; *B. longum*, *L. plantarum*, *L. salivarius*, and *L. rhamnosus* were tested for their antimicrobial activity against *R. solanacearum* (Fig. 1). All tested LAB-CFCS significantly inhibited the growth of *R. solanacearum* (Fig. 1a) with minimal inhibition recorded at the lowest volume (10 µL) of LAB-CFCS, whilst the highest volumes of LAB-CFCS exhibited significantly greater inhibition. Thus, the highest volume (50 µL) showed greater inhibition compared to lowest volumes (10, 20 and 30 µL) and the control with inhibition zones of 11.0, 9.9, 10.3, and 11.5 mm was recorded for *B. longum*, *L. plantarum*, *L. salivarius*, and *L. rhamnosus*, respectively (Fig. 1b). However, no significant effect was observed between 40 and 50 µL in *L. plantarum* and *L. rhamnosus.*

### Minimum inhibitory concentration (MIC) of lactic acid bacteria cell-free culture supernatants (LAB-CFCS)

**Fig. 2:**
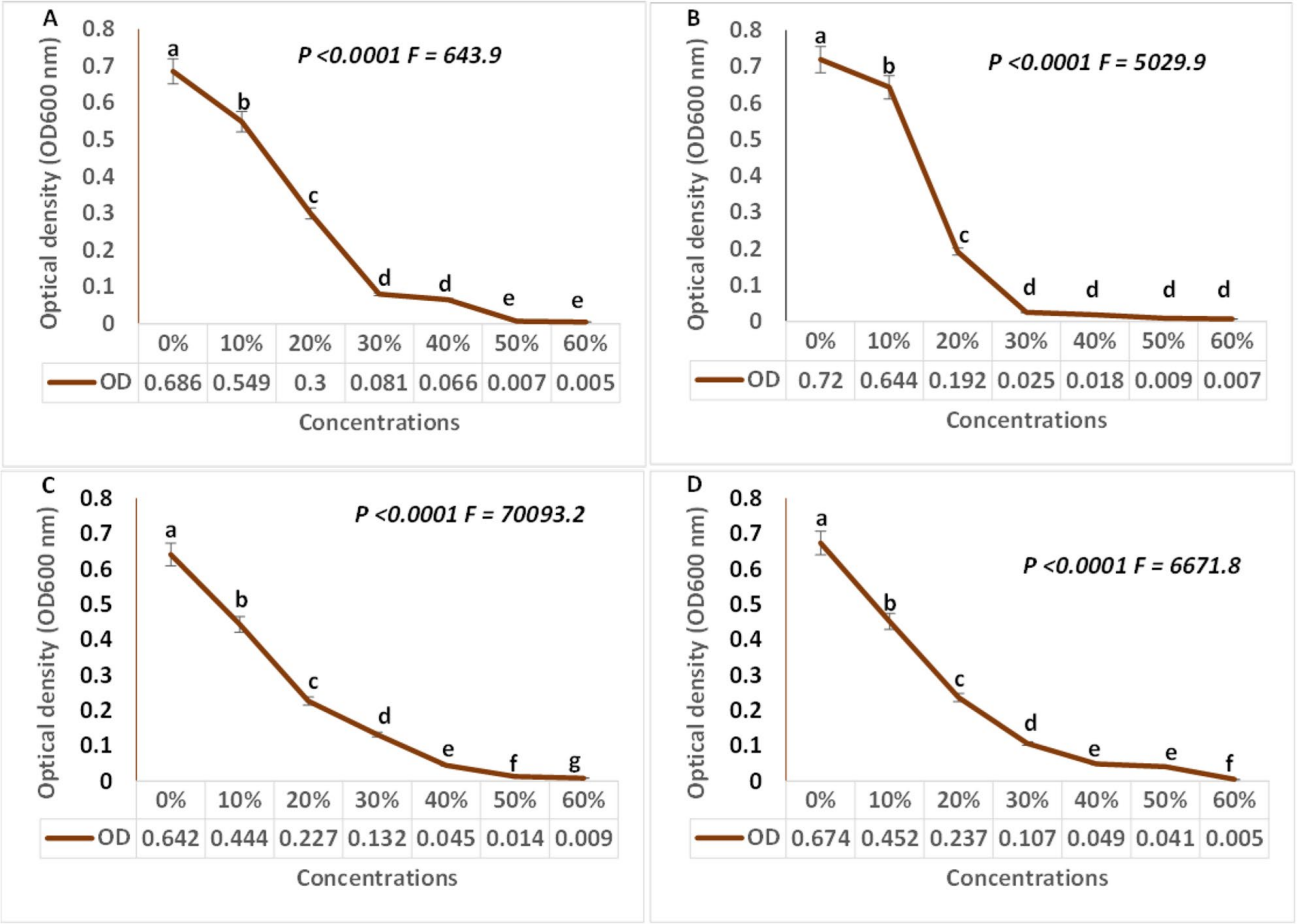
Minimum inhibitory concentration (MIC) of four lactic acid bacteria cell-free culture supernatants (LAB- CFCS) **A**) *B. longum*, **B**) *L. plantarum*, **C**) *L. salivarius*, and **D**) *L. rhamnosus.* The experiments were repeated twice independently. The data presented as means ± standard deviation of five biological replicates. Different letters indicate statistically significant differences among treatments, while the same letters signify no significant differences between them according to Tukey’s honestly significant difference test (*p* < 0.05)

The MIC showed variations among tested LAB-CFCS of lactic acid bacteria and their concentrations against *R. solanacearum* (Fig. 2). The MIC of CFCS from *B. longum* showed the highest bacterial growth inhibition represented by OD_600_ recording absorbances of 0.007 and 0.005 at the concentrations of 50 and 60%, respectively. There was no significant effect between the LAB-CFCS concentrations from 50 to 60%, which indicates that 50% was considered the MIC for *B. longum* (Fig. 2a). However, for *L. plantarum* the MIC was determined at 30% (OD_600_ = 0.025) showing no significant effect when compared to the higher concentrations of 40, 50 and 60% (Fig. 2b). The MIC of *L. salivarius* and *L. rhamnosus* was observed at 60%, which showed low bacterial growth inhibitions estimated at OD_600_ (0.009 and 0.005) with a significant inhibition compared to the lower concentrations (Fig. 2c, d).

### Inhibitory effect of lactic acid bacteria cell-free culture supernatants (LAB-CFCS) on biofilm formation of *R. solanacearum*

**Fig. 3:**
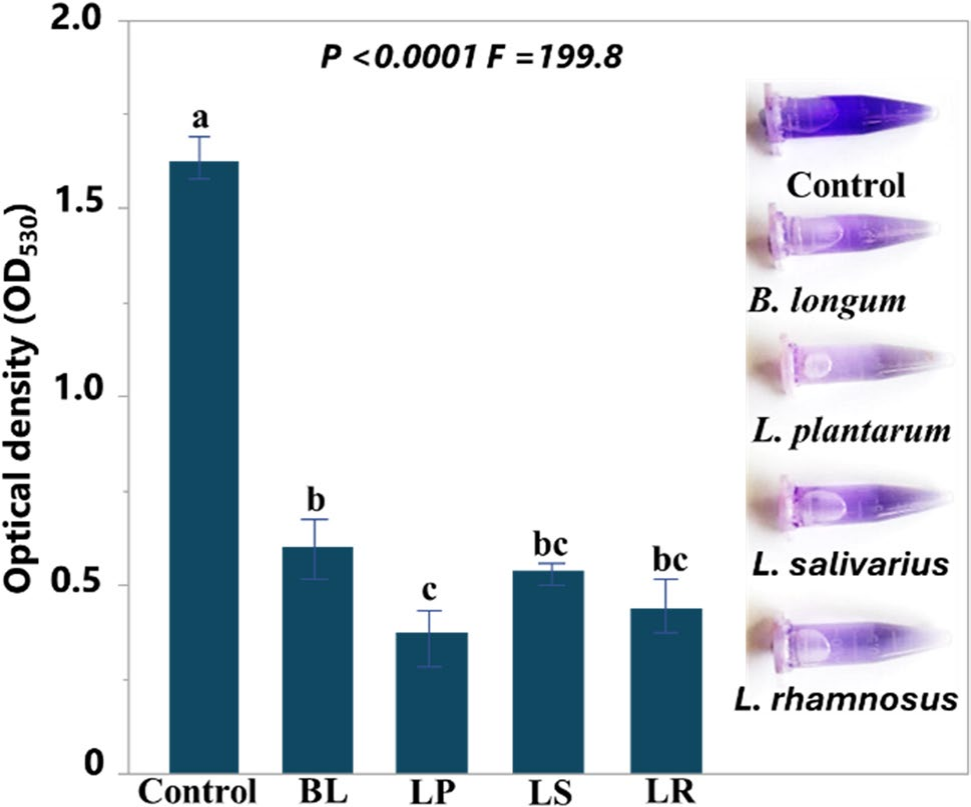
The influence of lactic acid bacteria cell-free culture supernatants (LAB-CFCS) on biofilm formation of *R. solanacearum*. BL) *B. longum*, LP) *L. plantarum*, LS) *L. salivarius*, and LR) *L. rhamnosus.* The experiments were repeated twice independently. The data presented as means ± standard deviation of five biological replicates. Different letters indicate statistically significant differences among treatments, while the same letters signify no significant differences between them according to Tukey’s honestly significant difference test (*p* < 0.05)

Biofilm formation by *R. solanacearum* was significantly affected by application of four LAB-CFCS, *B. longum*, *L. plantarum*, *L. salivarius*, and *L. rhamnosus* (Fig. 3). The untreated control recorded the highest value 1.62 at OD_530_ which indicates well formation of biofilm by *R. solanacearum.* In contrast, treating the bacteria with CFCS of *L. plantarum* (0.37), *L. rhamnosus* (0.44), *L. salivarius* (0.54) and *B. longum* (0.61) significantly inhibited the biofilm formation of *R. solanacearum* compared to the control (1.62). No significant differences was found among CFCS of *B. longum*,* L. salivarius* and *L. rhamnosus.*

### In vivo effect of lactic acid bacteria cell-free culture supernatants (LAB-CFCS) on tomato seedlings infected with *R. solanacearum*

**Fig. 4:**
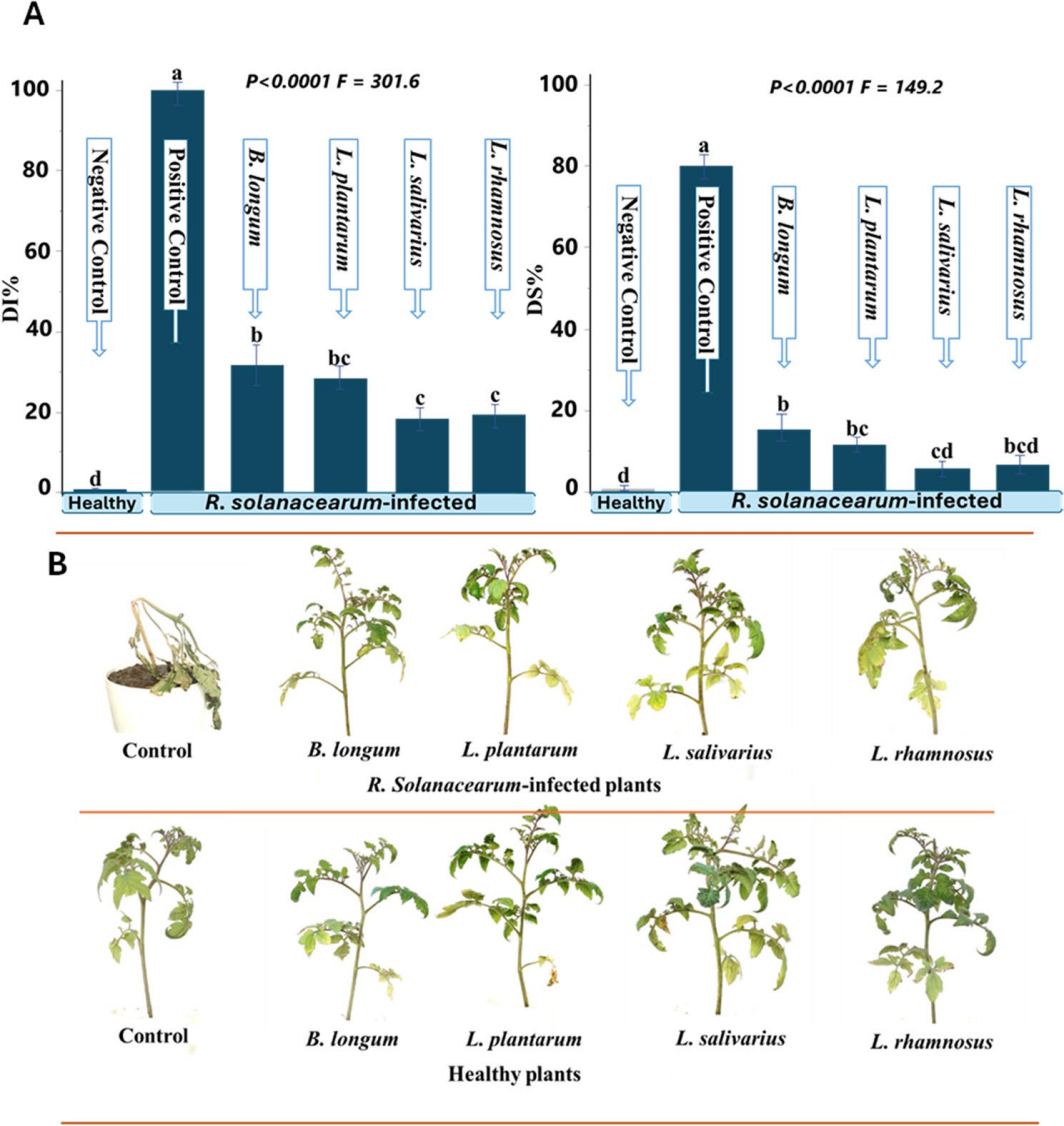
Effect of four lactic acid bacteria cell-free culture supernatants (LAB- CFCS): *B. longum*, *L. plantarum*, *L. salivarius*, and *L. rhamnosus* on the infection of tomato variety 054 F1 by *R. solanacearum.* The data in **A** show the influence of applied LAB-CFCS on disease incidence and disease severity. **B** Showing the impact of tested LAB-CFCS on healthy and infected tomato seedlings under greenhouse conditions. The data were recorded two weeks after inoculation with *R. solanacearum* and presented as means ± standard deviation of five replicates, each replicate represented by 10 pots/plants. Different letters indicate statistically significant differences among treatments, while the same letters signify no significant differences between them according to Tukey’s honestly significant difference test (*p* < 0.05). The experiment was repeated twice

The impact of applying cell-free culture supernatants derived from four lactic acid bacteria; *B. longum*, *L. plantarum*, *L. salivarius*, and *L. rhamnosus* was investigated in *R. solanacearum-*infected tomato plants through the assessment of wilt incidence and disease severity two weeks after pathogen inoculation (Fig. 4a, b). Wilting symptoms were observed in infected tomato seedlings that did not receive LAB-CFCS treatment. Initial symptoms appeared as wilting of the youngest leaves, followed by progressive leaf yellowing as disease development advanced (Fig. 4b). In addition, infected seedlings exhibited root rot symptoms and brown discoloration of the vascular tissues upon stem cutting.

When the freshly cut stem bases were immersed in water, a characteristic stream of white, slimy bacterial ooze was released, confirming *R. solanacearum* infection. These symptoms were consistent with the typical manifestations of bacterial wilt caused by *R. solanacearum*. In contrast, healthy non-inoculated tomato seedlings showed no visible wilt symptoms.

Application of LAB-CFCS significantly reduced the number of wilted seedlings compared to the untreated control. The lowest DI (19.0%) was observed when *R. solanacearum-*infected seedlings were treated with the CFCS of *L. salivarius*, followed by seedlings treated with *L. rhamnosus* (20.7%), no significant effect was noticed between these two bacterial CFCS. In addition, the CFCS of *L. plantarum* and *B. longum* significantly alleviated wilt incidence produced by *R. solanacearum* to be 31.7 and 28.3% compared to 100% for the control. Furthermore, the tested LAB-CFCS significantly alleviated wilt symptoms compared to the untreated infected control. The highest wilt severity (80.0%) was recorded on the infected untreated tomato plants. However, treating the infected tomato seedlings with *L. salivarius*,* L. rhamnosus*, *L. plantarum*, and *B. longum* significantly reduced wilt severity compared to infected plants with severity percentages of 5.0, 6.7, 11.7 and 14.7%, respectively.

### Production of lactic and acetic acids by four lactic acid bacteria

**Fig. 5:**
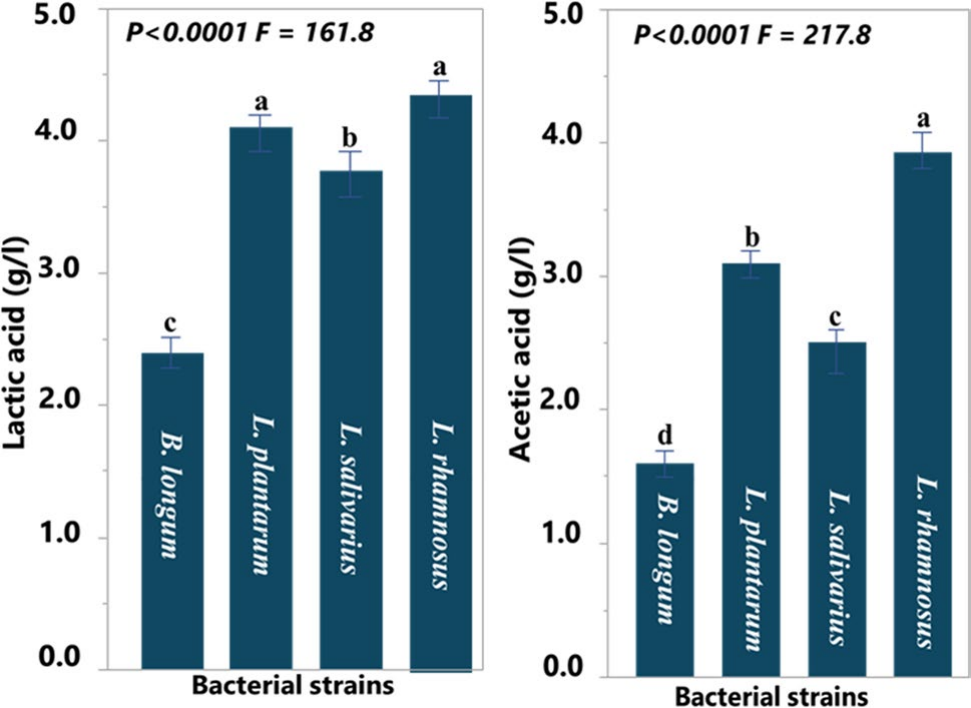
Quantification of lactic and acetic acids in four lactic acid bacteria cell-free culture supernatants (LAB-CFCS). The data presented as means ± standard deviation of five replicates. Different letters indicate statistically significant differences among treatments, while the same letters signify no significant differences between them according to Tukey’s honestly significant difference test (*p* < 0.05)

The ability of four lactic acid bacteria to produce lactic and acetic acid was estimated by measuring the concentration of both acids in the CFCS of each bacterium (Fig. 5). The production of lactic and acetic acids was confirmed in the four LAB-CFCS, but the bacteria varied in their ability to produce the acids. The highest levels of lactic acid (4.4 and 4.1 g/L) were produced by *L. rhamnosus* and *L. plantarum*, respectively, which were significantly higher than the amount produced by *L. salivarius.* However, the lowest lactic acid concentration (2.4 g/L) was produced by *B. longum.* A similar trend was observed when measuring the concentration of acetic acid, which *L. rhamnosus* produced a significantly higher amount of acetic acid (3.9 g/L) compared to the other bacteria followed by *L. plantarum* (3.1 g/L), while *B. longum* produced the lowest amount of acetic acid (1.6 g/l).

### Physiological traits

#### Photosynthetic pigments and organic acids

**Fig. 6:**
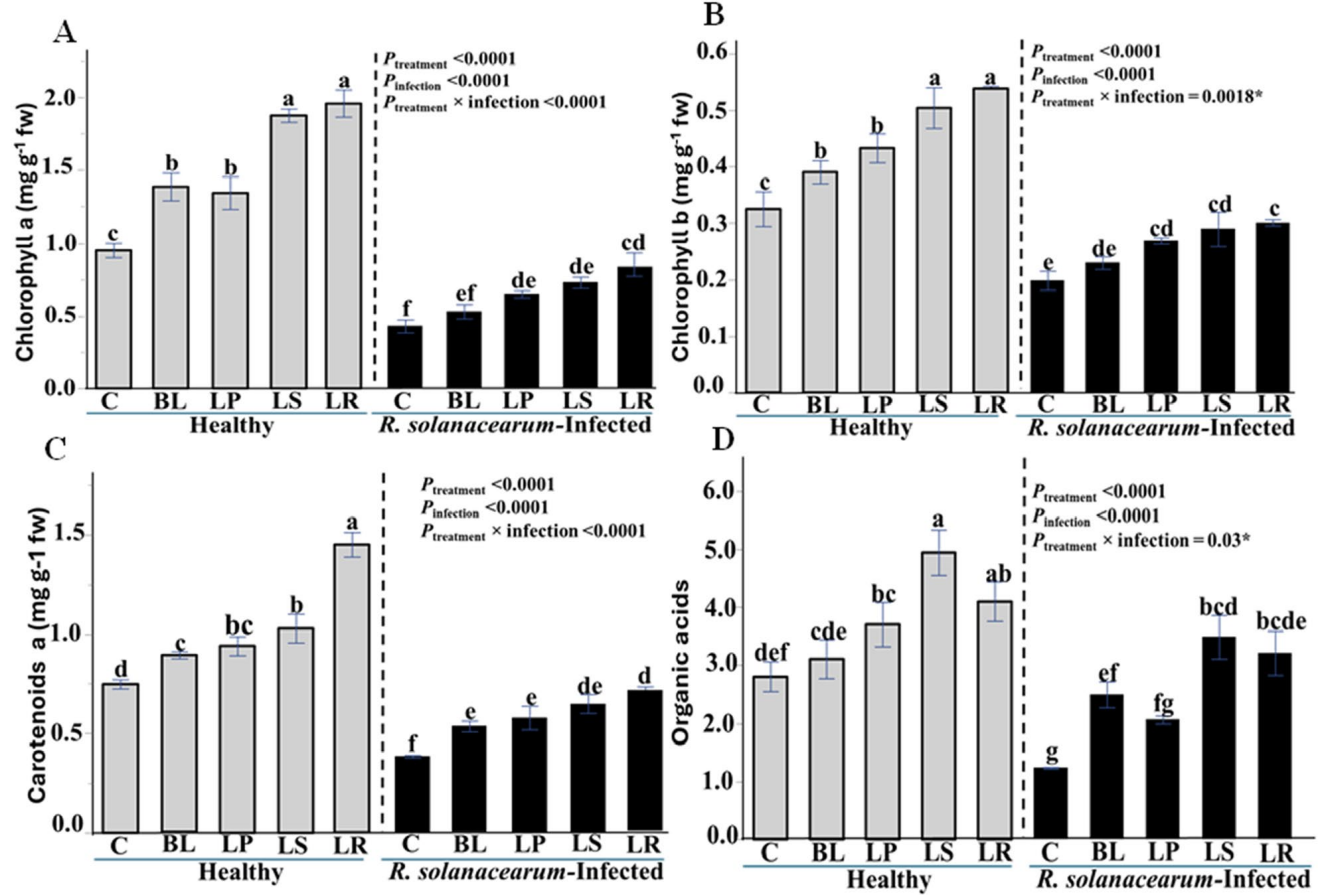
The impact of applying lactic acid bacteria cell-free culture supernatants (LAB- CFCS) to healthy and *R. solanacearum*-infected tomato seedlings on plant pigments; **A**. chlorophyll a, **B**. chlorophyll b, **C**. carotenoids and **D**. organic acids content. BL) *B. longum*, LP) *L. plantarum*, LS) *L. salivarius*, and LR) *L. rhamnosus.* The experiments were repeated twice independently. The data presented as means ± standard deviation of five biological replicates. Different letters indicate statistically significant differences among treatments, while the same letters signify no significant differences between them according to Tukey’s honestly significant difference test (*p* < 0.05)

Infection with *R. solanacearum* significantly reduced the contents of chlorophyll *a*, chlorophyll *b*, carotenoids, and organic acids in tomato seedlings by 54.63%, 39.08%, 48.5%, and 57.2%, respectively, compared with the healthy untreated control. However, all LAB-CFCS treatments increased chlorophylls and carotenoids content in both healthy and infected plants, with the greatest improvement observed under *L. rhamnosus* treatment. (Fig. 6a, b,c). Organic acids are negatively affected by the infection with *R. solanacearum*. In *R. solanacearum*-infected treated seedlings, all LAB-CFCS treatments significantly increased organic acid levels in healthy plants compared to the control except for *L. plantarum* where the difference was not significant (Fig. 6d). The highest increase was recorded in *L. salivarius*, which showed a 92.3% increase over control.

#### Secondary metabolites

**Fig. 7:**
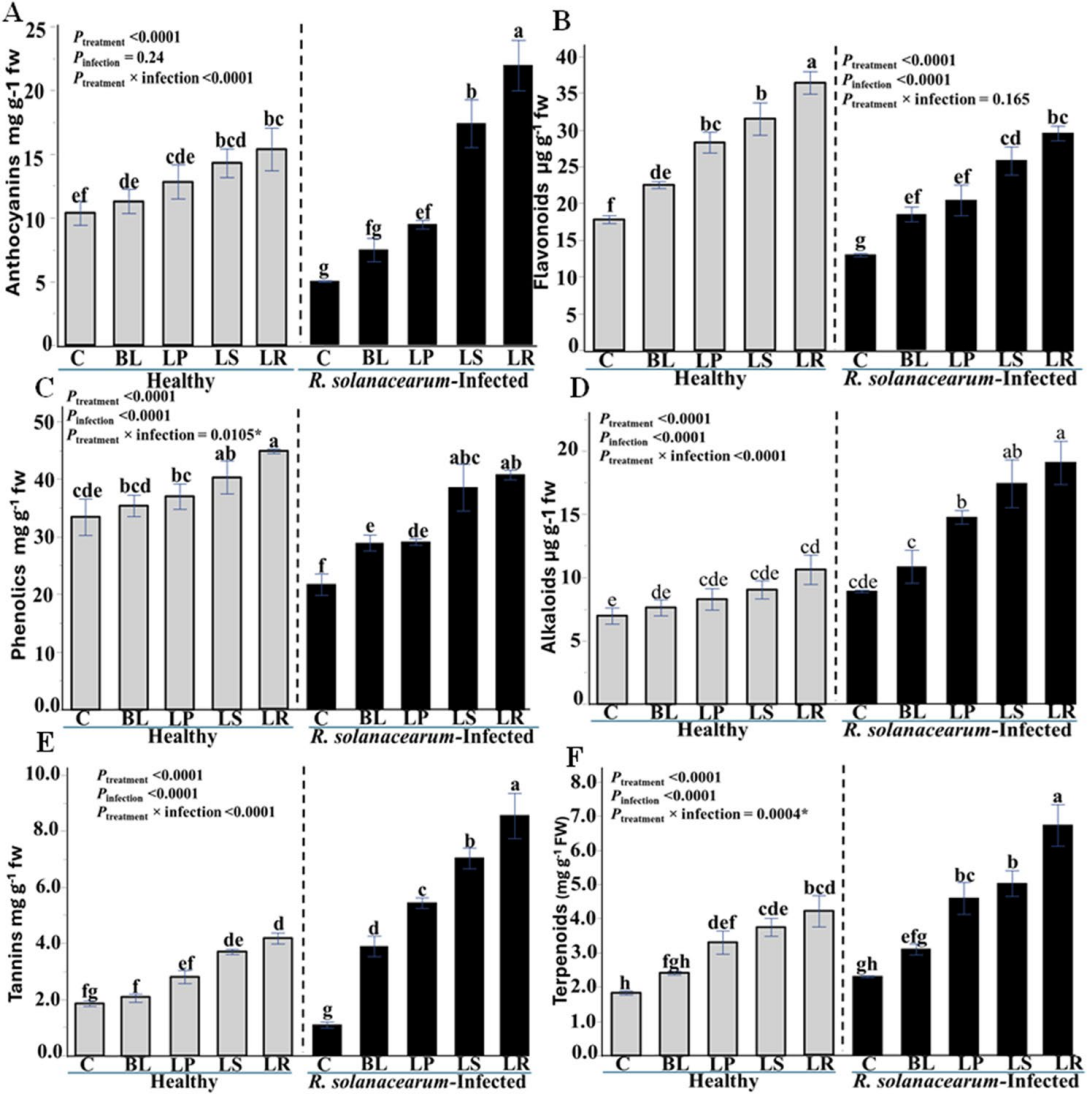
Effect of applying lactic acid bacteria cell-free culture supernatants (LAB-CFCS) to healthy- and *R. solanacearum*-infected tomato seedlings on plant pigment; **A**. Anthocyanins, **B**. flavonoids, **C**. phenolics, **D**. alkaloids, (**E**) tannins and (**F**) terpenoids content. BL) *B. longum*, LP) *L. plantarum*, LS) *L. salivarius*, and LR) *L. rhamnosus.* The experiments were repeated twice independently. The data presented as means ± standard deviation of five biological replicates. Different letters indicate statistically significant differences among treatments, while the same letters signify no significant differences between them according to Tukey’s honestly significant difference test (*p* < 0.05)

In tomato seedlings, the accumulation of secondary metabolites, such as anthocyanins, flavonoids, phenolics, alkaloids, tannins, and terpenoids, was influenced by *R. solanacearum infection* and LAB-CFCS application (Fig. 7). Unlike *B. longum* and *L. plantarum*, application of CFCS of *L. salivarius* and *L. rhamnosus* significantly increased the tomato content of anthocyanin in healthy and infected plants compared to the controls (Fig. 7a). The highest anthocyanin content (21.9 mg g⁻¹ fresh weight) was observed in *R. solanacearum*-infected tomato seedlings treated with the CFCS of *L. rhamnosus*. In the untreated controls, infection with *R. solanacearum* significantly decreased flavonoid, phenolic, and tannin contents compared to the healthy control, with reductions of 27.17%, 35.13%, and 41.3%, respectively (Fig. 7b, c,e). In healthy plants, supplementation with LAB-CFCS increased the levels of secondary metabolites, with *L. plantarum* and *L. rhamnosus* showing significantly higher accumulation. Under infection, all LAB-CFCS treatments mitigated the declines in flavonoids, phenolics, and tannins relative to the infected control. Among them, *L. rhamnosus* showed the most pronounced effect, highlighting its superior protective capacity and its strong stimulation of secondary metabolite biosynthesis.

In contrast to the previously mentioned secondary metabolites, alkaloids and terpenoids exhibited an opposite trend under infection. Infection with *R. solanacearum* insignificantly increased alkaloid and terpenoid levels compared with healthy plants, indicating the activation of defense-related metabolic pathways. In healthy plants, LAB-CFCS treatments slightly raised alkaloid levels.

In infected plants, all LAB-CFCS treatments except *B. longum* resulted in a significant and pronounced accumulation of alkaloids and terpenoids, surpassing both the healthy and infected controls (Fig. 7d, f). This highlights the synergistic effect of LAB-CFCS in priming the plants for enhanced secondary metabolite production under pathogen stress. The data also revealed that *R. solanacearum* infection stimulates some i.e. secondary metabolite production as part of the plant’s defense response. LAB-CFCS treatment, particularly with *L. rhamnosus* and *L. plantarum*, amplifies this effect, indicating their role in boosting host resistance and metabolic reprogramming.

#### Oxidizing agents and oxidative stress

**Fig. 8:**
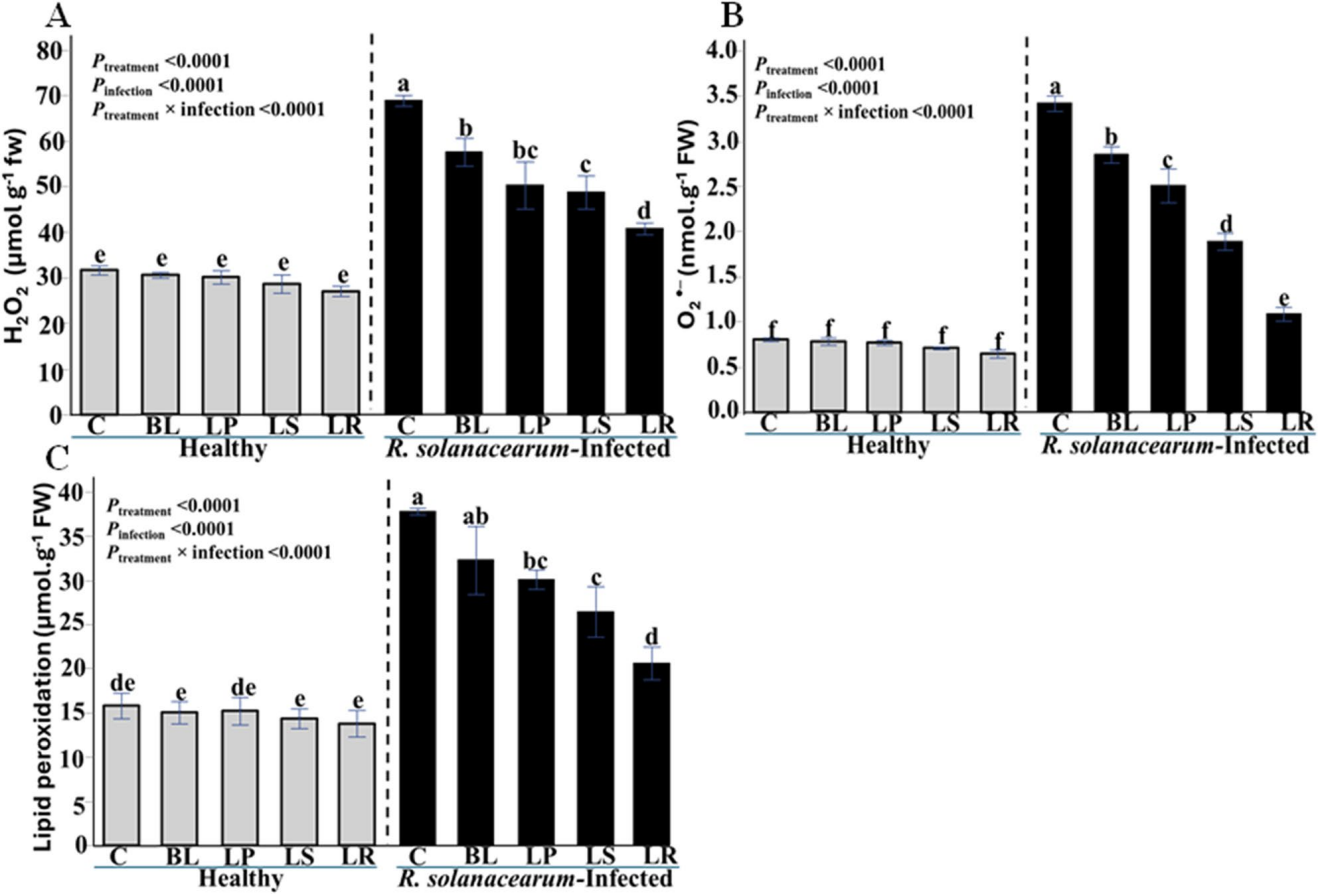
Effect of applying lactic acid bacteria cell-free culture supernatants (LAB- CFCS) to healthy- and *R. solanacearum*-infected tomato seedlings on oxidizing agents and oxidative stress: **A** Hydrogen peroxide H_2_O_2_, **B** Superoxide anion (O_2_^•─^) and **C** Lipid peroxidation. BL) *B. longum*, LP) *L. plantarum*, LS) *L. salivarius*, and LR) *L. rhamnosus.* The experiments were repeated twice independently. The data presented as means ± standard deviation of five biological replicates. Different letters indicate statistically significant differences among treatments, while the same letters signify no significant differences between them according to Tukey’s honestly significant difference test (*p* < 0.05)

The levels of oxidizing agents and oxidative stress (H₂O₂, O₂·⁻, and lipid peroxidation) were significantly influenced by *R. solanacearum* infection, LAB-CFCS treatments, and their interaction (Fig. 8). Infection with *R. solanacearum* strongly increased all oxidative stress indicators (*P* < 0.0001). Infected control plants accumulated the highest levels of H₂O₂ (~ 69 µmol g⁻¹ FW), O₂·⁻ (~ 3.4 µmol g⁻¹ FW), and lipid peroxidation (~ 37.8 µmol g⁻¹ FW), confirming severe oxidative damage under pathogen pressure. In healthy plants, application of LAB-CFCS showed only minor differences compared with the untreated control, maintaining relatively low concentrations of H₂O₂ (≈ 28–31.7 µmol g⁻¹ FW) (Fig. 8a), O₂·⁻ (≈ 0.5–0.8 µmol g⁻¹ FW) (Fig. 8b), and lipid peroxidation (≈ 13.7–15.8 µmol g⁻¹ FW) (Fig. 8c). However, in infected plants, the application of LAB-CFCS markedly alleviated oxidative. Among all LAB-CFCS, *L. rhamnosus* and *L. plantarum* were the most effective which significantly reduced the accumulation of H₂O₂ and O₂·⁻ as well as lipid peroxidation compared with untreated-infected controls. For instance, *L. rhamnosus* lowered the levels of H₂O₂ to ~ 48.8 µmol g⁻¹ FW and O₂·⁻ to ~ 1.9 µmol g⁻¹ FW, while lipid peroxidation was decreased to 26.4 µmol g⁻¹ FW.

#### Signaling molecules

**Fig. 9:**
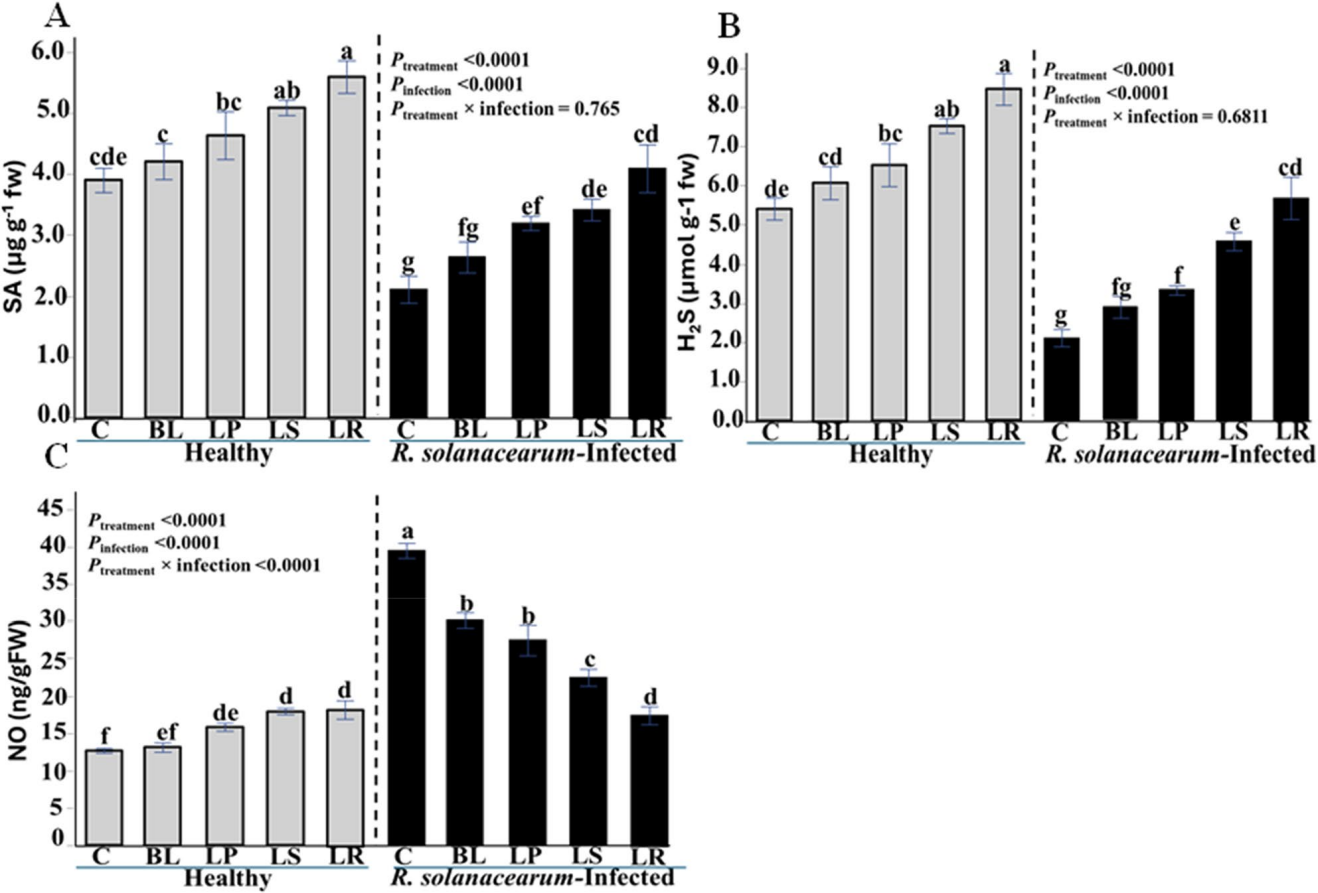
The effect of applying lactic acid bacteria cell-free culture supernatants (LAB- CFCS) to healthy- and *R. solanacearum*-infected tomato seedlings on signaling molecular markers: **A** Salicylic acid (SA), **B** Hydrogen sulfide (H_2_S) and **C** Nitric oxide (NO). BL) *B. longum*, LP) *L. plantarum*, LS) *L. salivarius*, and LR) *L. rhamnosus.* The experiments were repeated twice independently. The data presented as means ± standard deviation of five biological replicates. Different letters indicate statistically significant differences among treatments, while the same letters signify no significant differences between them according to Tukey’s honestly significant difference test (*p* < 0.05)

The infection by *R. solanacearum* markedly reduced the accumulation of SA and H₂S compared with healthy plants, which indicates a shortage in defense responses against infection. LAB-CFCS treatment restored the reduction in both molecules. In *R. solanacearum*-infected seedlings, the application of *L. rhamnosus* and *L. plantarum* expressed the highest contents of SA (4.1 and 3.4) and H_2_S (4.6 and 5.7 µg g⁻¹ FW), compared to 2.1 and 1.8 µg g⁻¹ FW in infected-untreated control, respectively (Fig. 9a, b).

Unlike SA and H_2_S, the infection by *R. solanacearum* significantly increased the seedling content of NO which reached 12.7 ng g⁻¹ FW in healthy control compared to 39.5 ng g⁻¹ FW in the infected untreated control (Fig. 9c). LAB-CFCS application maintained NO levels above those of the control in treated healthy plants, while it variably reduced NO biosynthesis in infected plants; among the treatments, *L. rhamnosus* recorded the lowest value (17.6 ng g⁻¹ FW) compared with the infected untreated control (39.5 ng g⁻¹ FW). Comparing the influence of LAB-CFCS application on the level of NO in healthy and infected tomato seedlings, the results indicated that the CFCS of *L. rhamnosus* was the only treatment that kept the NO at a similar level to healthy seedlings under the same treatment.

#### Antioxidant enzymes and secondary metabolites-related enzymes

**Fig. 10:**
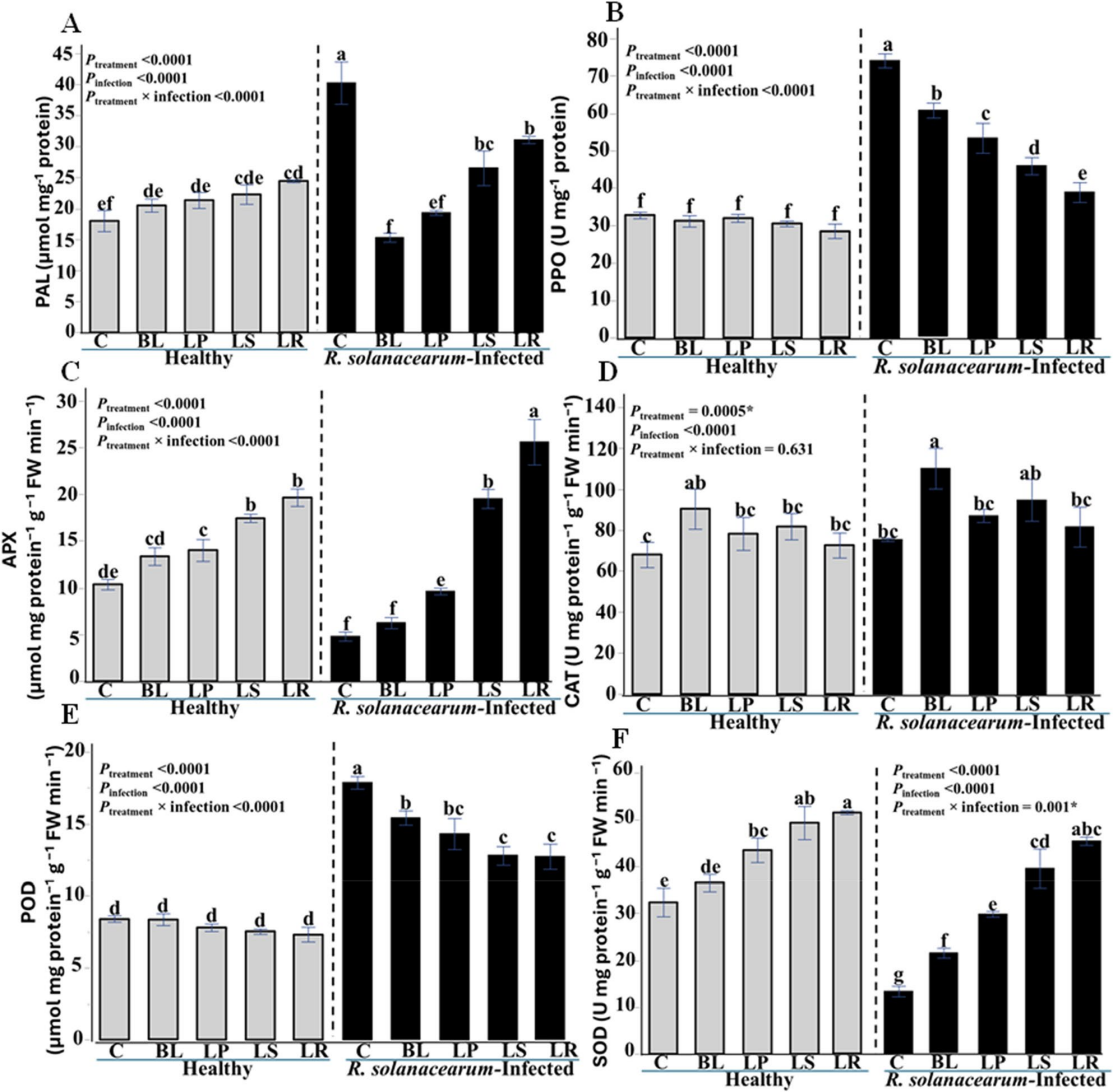
Effect of applying lactic acid bacteria cell-free culture supernatants (LAB-CFCS) to healthy- and *R. solanacearum*-treated tomato seedlings on antioxidant enzymes and secondary metabolites-related enzymes; **A** Phenylalanine Ammonia-Lyase (PAL), **B** Polyphenol oxidase (PPO), **C** Ascorbate peroxidase (APX), **D** Catalase (CAT) **E** Guaiacol peroxidase (POD) and **F**, Superoxide dismutase (SOD). BL) *B. longum*, LP) *L. plantarum*, LS) *L. salivarius*, and LR) *L. rhamnosus.* The experiments were repeated twice independently. The data presented as means ± standard deviation of five biological replicates. Different letters indicate statistically significant differences among treatments, while the same letters signify no significant differences between them according to Tukey’s honestly significant difference test (*p* < 0.05)

In infected untreated control, the enzyme involved in secondary metabolite synthesis enhanced the activity of PAL, which increased by 123.447% compared to healthy untreated control. In infected seedlings, the application of LAB-CFCS retarded the activity of PAL, especially in seedlings treated with CFCS of *B. longum* and *L. plantarum* (Fig. 10a). Polyphenol oxidase (PPO) activity was strongly induced by infection compared to healthy control. LAB-CFCS application significantly decreased the accumulation of PPO in infected plants compared to the infected untreated control. In healthy plants, no significant impact was observed between the level of PPO in treated and control seedlings (Fig. 10b). The infection by *R. solanacearum* significantly decreased the activities of ascorbate peroxidase and superoxide dismutase by 53.7 and 58.7% compared in relative to the healthy control, respectively (Fig. 10c, f). However, LAB-CFCS treatments, especially *L. rhamnosus* and *L. plantarum*, significantly enhanced the activities of APX and SOD in infected seedlings.

No significant changes were observed in the activity of catalase (CAT) between the infected and healthy controls. In healthy and infected seedlings LAB-CFCS slightly increased the activity of CAT with only *B. longum* showed a significant difference (Fig. 10d). The activity of peroxidase (POD) increased significantly due to the stress resulted by *R. solanacearum* infection compared to healthy plants recording a 112% increase compared to healthy control. Although LAB-CFCS treatments showed a non-significant impact on the activity of POD in healthy seedlings, it demonstrated a significant influence on the activity of POD in infected seedlings. LAB-CFCS decreased the level of POD in infected seedlings compared to the infected untreated control. *L. salivarius and L. rhamnosus* achieved the lowest POD values (7.5 and 7.3 µmol mg protein^**− 1**^ g^**− 1**^ FW min^**− 1**^) comparing with the infected untreated control (17.9 µmol mg protein^**− 1**^ g^**− 1**^ FW min^**− 1**^) (Fig. 10e).

#### Heatmap and principal component analysis

**Fig. 11:**
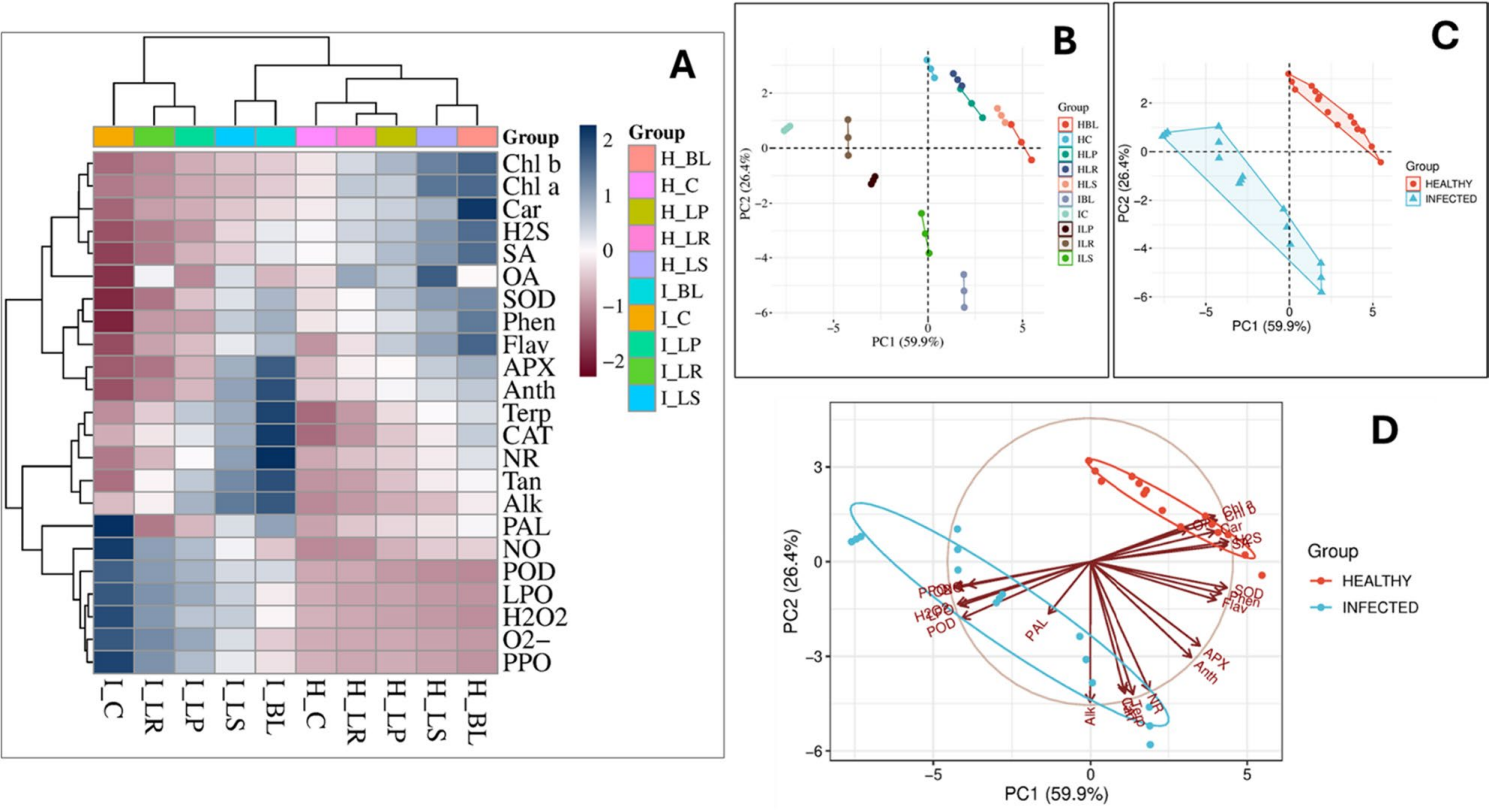
**A** Heatmap with hierarchical clustering illustrates the metabolic and biochemical responses of tomato seedlings under different treatments. Treatments include healthy controls (H_C), infected controls (I_C), and plants treated with LAB-CFCS derived from *B. longum* (BL), *L. plantarum* (LP), *L. salivarius* (LS), and *L. rhamnosus* (LR), applied either to healthy (H_BL, H_LP, H_LS, H_LR) or infected plants (I_BL, I_LP, I_LS, I_LR). **B** PCA score plots separating all treatment groups, demonstrating clear differences between healthy and infected plants and the effect of each LAB-CFCS on tomato metabolism. **C** PCA grouping of healthy vs. infected plants illustrating distinct metabolic/biochemical profiles induced by infection and the modulatory effect of CFCS application. **D** PCA biplot showing variables and their contribution to different treatments. Antioxidant enzymes (POD, APX, CAT), phenolics, flavonoids, and stress markers (H₂O₂, O₂•⁻, MDA) drive the divergence between infected and healthy plants, while LAB-CFCS treatments shift infected seedlings toward healthier biochemical signatures

The heatmap (Fig. 11a) shows distinct metabolite correlations between healthy and infected seedlings, with infected plants displaying intensified oxidative markers (H₂O₂, O₂•⁻, and LPO) and suppression of antioxidant enzymes. LAB-CFCS treatments, especially *L. plantarum* and *L. rhamnosus*, shifted infected plants closer to the healthy biochemical traits, reducing oxidative stress markers and enhancing phenolics, flavonoids, SOD, CAT, APX, and other defense-related metabolites. Principal component analysis (PCA) of all treatment groups (Fig. 11b) demonstrates clear separation between healthy and infected seedlings along PC1 (59.9% variance). Infected plants were grouped far from healthy controls due to elevated stress metabolites. CFCS-treated infected seedlings shifted toward the healthy cluster, indicating partial restoration of metabolic balance. The *L. plantarum* and *L. rhamnosus* CFCS showed the strongest restorative effect. The PCA analysis presented in Figure (11c), shows healthy plants clustered tightly, whereas infected plants form a distant, more dispersed group due to the high variability induced by the disease stress. In Figure (11d), the biplot representation of PCA reveals that stress markers (H₂O₂, O₂•⁻, LPO, POD) strongly correlate with infected plants. Defense-related metabolites such as total phenolics, flavonoids, antioxidants, APX, CAT, terpenoids, alkaloids, and anthocyanins are strongly associated with healthy and LAB-CFCS-treated plants. The directional vectors show that CFCS, especially from *L. plantarum* and *L. rhamnosus*, enhance biochemical components linked to stress resistance.

## Discussion

In this study, the cell-free culture supernatants of four lactic acid bacteria (LAB) strains, namely *B. longum*, *L. plantarum*, *L. salivarius*, and *L. rhamnosus* were evaluated for their antibacterial efficacy against *R. solanacearum*, as well as their effects on the vegetative growth and physiological performance of tomato plants. The in vitro results demonstrated that the CFCS of all tested lactic acid bacterial strains significantly suppressed the growth of *R. solanacearum* and effectively inhibited bacterial biofilm formation. Moreover, the inhibition zones increased markedly with increasing volumes of LAB-CFCS, indicating a dose-dependent antibacterial effect.

The minimum inhibitory concentration varied among the tested LAB strains, reaching 50% for *B. longum*, 30% for *L. plantarum*, and 60% for both *L. salivarius* and *L. rhamnosus*. The antimicrobial potential of LAB against a wide range of phytopathogenic fungi [25, 27, 64–66] and bacteria [19, 28, 31, 67] has been extensively documented.

Our study verified that all tested LAB strains could produce organic acids, primarily lactic and acetic acids. Notably, *L. plantarum*, *L. salivarius*, and *L. rhamnosus* produced higher concentration organic acids compared to *B. longum*. Wang et al., [30] denoted that the production of lactic and acetic acid lowers the pH, which inhibits fungal/bacterial growth. Although *R. solanacearum* can tolerate and grow under mildly acidic conditions, its growth is is severely inhibited at pH levels below 4.0 [68, 69]. Thus, the pronounced acidity (≤ 4.0) induced by LAB-CFCS contributed substantially to the inhibition of *R. solanacearum* growth under in vitro conditions. In addition to organic acids production, the antibacterial activity of LAB-CFCS may also be attributed to the synthesis of bacteriocins such as plantaricin, which disrupt bacterial cell membranes and ultimately induce cell death, and hydrogen peroxide that collectively suppress fungal and bacterial pathogens [30, 70, 71]. Additionally, LAB are known to produce several other bioactive antimicrobial compounds, including diketopiperazines, hydroxy derivatives of fatty acids, 3-phenyllactate, bacteriocins and bacteriocin-like inhibitory substances (BLIS), pyrrolidone-5-carboxylic acid, diacetyl, and reuterin [71–73]. Moreover, studies by Kwak et al., [74] and Vougiouklaki et al., [75] reported that LAB-derived metabolites, such as hydrogen peroxide, acetate, butyrate, formic acid, succinate, propionate, valerate, caproic acid, and cyclic dipeptides exhibit strong antimicrobial activities against diverse microbial pathogens.

A significant reduction in wilt incidence and severity was observed when CFCS was applied to *R. solanacearum*-infected tomato seedlings. Many studies have confirmed the efficacy of LAB in mitigating plant bacterial diseases [20, 28, 31]. In the present study, the effectiveness of LAB-CFCS treatments on both healthy and *R. solanacearum*-infected plants was assessed by analyzing key physiological parameters. Across the four LAB, the CFCS of *L. rhamnosus*, demonstrated significant enhancements, especially under infection stress conditions. Chlorophyll degradation is a common symptom of *R. solanacearum* infection [76]. The pronounced decline in chlorophyll a, chlorophyll b, and carotenoids under *R. solanacearum* infection reflects the detrimental impact of bacterial wilt on the photosynthetic machinery of plants. Pathogen infection often accelerates chlorophyll degradation and impairs pigment biosynthesis, resulting in reduced light-harvesting efficiency and compromised photosynthetic performance [77, 78].

In addition, wilting and yellowing of leaves induced from the blockage of xylem vessels by bacterial biofilms, severely restricting water and nutrient flow. This blockage not only leads to dehydration but also impairs photosynthesis, contributing to reduced growth and vitality of the plant [79]. However, the observed decrease in carotenoids under infection by *R. solanacearum* further suggests weakened photoprotection, as carotenoids play a critical role in quenching ROS generated during pathogen stress [80]. Interestingly, heatmap and PCA analyses revealed that LAB-CFCS application significantly mitigated the infection by *R. solanacearum* and enhanced pigment contents in both healthy and infected plants. Among the tested strains, *L. rhamnosus* exhibited the most pronounced effect, suggesting effective protection of the photosynthetic apparatus. The restoration of chlorophyll and carotenoid levels indicates that the culture filtrates of the LAB may enhance host metabolism by producing phytohormones (e.g., IAA, cytokinins), promoting nutrient availability by modulating the uptake of important nutrients like phosphorus and potassium, fixing nitrogen, and the production of plant hormones and siderophores as well as activating stress-mitigating pathways [81, 82]. In addition, LAB-CFCS-mediated suppression of pathogen growth could indirectly reduce oxidative damage, thereby sustaining chlorophyll stability under infection. Several LAB species, such as *L. plantarum*, have been reported to secrete phytohormones including gibberellins (GA) and auxins such as indole-3-acetic acid (IAA), which are crucial for plant growth and development [72, 83].

The reduction in organic acids under pathogen stress reflects a disruption in primary metabolism, as organic acids are central intermediates of the tricarboxylic acid (TCA) cycle and function as signaling molecules in plant defense [84]. This response is associated with a decline in photosynthetic pigments and energy supply, which contributes to reduced growth and impaired defense responses [82]. LAB-CFCS treatments effectively mitigated this decline, with the most striking effect recorded in *L. salivarius* for healthy and *R. solanacearum*-infected plants. These results suggest that LAB may boost organic acid biosynthesis by stimulating microbial-plant interactions that enhance metabolic flux through the TCA cycle and related pathways and serve as stored forms of carbon formed during the conversion of carbon assimilates into vital metabolic pathways [85]. Increased organic acid levels may also improve root exudation, strengthening plant–microbe symbiosis, nutrient mobilization, and pathogen suppression in the rhizosphere. Also, elevated levels of organic acids metabolism under LAB induces programmed cell death in plant tissue which could regulate ROS homeostasis [86], which in turn triggers a cascade of signal transduction events leading to activation of plant defense against *R. solanacearum* infection. Additionally, the acidity generated by LAB through the production of organic acids contributes to the solubilization of phosphorus and potassium, thereby enhancing their availability for plant uptake [81]. This aligns with the role of LAB in modulating organic acid production to combat pathogenic infections.

The response of host plants to *R. solanacearum* involves multiple stress-related pathways, particularly the strong activation of reactive oxygen species (ROS). While ROS, at controlled levels, are essential defense signals that help reinforce cell walls and restrict pathogen invasion, their excessive accumulation leads to oxidative stress and cellular damage [87]. In this study, infection by *R. solanacearum* caused marked increases in H₂O₂, O₂·⁻, and lipid peroxidation, indicating severe oxidative stress and membrane destabilization. PCA and heatmap analyses showed that LAB-CFCS application, especially *L. rhamnosus* and *L. plantarum*, significantly reduced ROS build-up in *R. solanacearum*-infected seedlings and supported more balanced defense metabolites and signaling pathways. Jaffar et al. [81] also reported that LAB contribute to disease suppression via antimicrobial compounds, supporting their role in plant protection, induced systemic resistance (ISR) by priming jasmonic acid (JA) and salicylic acid (SA) pathways [88]. The results demonstrate that LAB-CFCS supplementation significantly influenced the accumulation of anthocyanins and alkaloids, indicating that the pathogen acts as a strong elicitor of secondary metabolism and nitrogen-containing metabolites (e.g., alkaloids) which are part of the plant’s innate immune response. These compounds aim to localize the infection, preventing its spread to healthier tissues [89], which recommend the results of present study.

A significant increase of SA in LAB-CFCS-treated seedlings compared to untreated infected plants was obviously detected in this study, which confirms that the CFCS of the tested LAB induced the resistance of plants against *R. solanacearum*. In addition to ROS, hormonal changes, particularly alterations in stress-related hormones like SA, JA, and ethylene, play a crucial role in a plant’s response to infection, with SA associated with systemic acquired resistance against biotrophic pathogens, while JA and ethylene are linked to responses against necrotrophic pathogens and wound healing [90]. Thus, the enhancement of SA levels is especially significant, as it implies activation of SAR, leading to stronger local and systemic resistance.

The observed reduction in H₂S under *R. solanacearum* infection indicates a compromised defense signaling capacity of the host. H₂S functions as a signaling gasotransmitter that interacts with SA, ROS, and NO to fine-tune stress responses [91]. This suppression in infected plants reflects the pathogen’s ability to interfere with or suppress host defense signaling, leaving plants more vulnerable to oxidative damage and pathogen colonization. LAB-CFCS treatments successfully restored H₂S levels, particularly with *L. rhamnosus* and *L. plantarum*. This recovery suggests that LAB can induce plant immunity by stimulating endogenous defense signaling pathways and may contribute to redox homeostasis and modulation of defense-related gene expression.

Unlikely, the infection caused a marked increase in NO accumulation, nearly tripling its levels compared with healthy controls. While NO is an important signaling molecule in defense, excessive accumulation can lead to nitrosative stress, protein modification (S-nitrosylation), and disruption of cellular homeostasis [77], thereby uncontrolled NO production, contributing to cellular injury and aiding pathogen progression. LAB-CFCS treatments mediated this effect that NO remained elevated in healthy LAB-CFCS-treated plants, while they reduced NO accumulation in infected plants. This balancing effect, most pronounced in *L. rhamnosus*, suggests that LAB-CFCS help in regulating NO biosynthesis, preventing its excessive accumulation while maintaining its signaling role in immunity. LAB-CFCS application, particularly with *L. rhamnosus* and *L. plantarum*, appeared to modulate this disrupted signaling network. By restoring SA and H₂S levels, LAB-CFCS reestablished their synergistic cooperation, which likely contributed to activating SAR and bolstering antioxidant defenses. LAB-CFCS moderated NO levels in infected plants, preventing its uncontrolled accumulation while maintaining it above basal levels in healthy plants. Thus, the findings of present study suggested that LAB-CFCS modulation of SA–H₂S–NO act as potentially associated signaling components. This not only alleviates oxidative stress but also enhances systemic immunity, contributing to improved plant resilience against *R. solanacearum*. Further molecular and genetic investigations are required to elucidate the precise cross-talk mechanisms among these signaling pathways.

The present findings demonstrate that *R. solanacearum* infection triggered significant changes in the antioxidant defense machinery of plants, as reflected in the modulation of POD, CAT, APX, and PPO activities. Infected plants generally exhibited elevated POD and PPO activities, which are closely linked to lignin formation and the reinforcement of cell walls, thereby restricting pathogen spread [92]. The strong induction of these enzymes in infected plants suggests that they play a central role in the defense responses to bacterial wilt [89]. However, infection also caused a decline in APX activity, indicating a disruption in the ascorbate glutathione cycle, which is essential for maintaining redox homeostasis under oxidative stress. This reduction could compromise the efficient scavenging of hydrogen peroxide, highlighting the pathogen’s ability to weaken certain components of the antioxidant system. The application of LAB-CFCS significantly enhanced the activity of all tested enzymes, especially in infected plants. The restoration and upregulation of CAT and APX activities under LAB-CFCS treatments indicate that these beneficial microbes can alleviate oxidative damage by promoting enzymatic detoxification of ROS and recommended by PCA and heatmap analyses. This effect likely contributes to reduced cellular injury and improved stress resilience. However, the POD and PPO activities slowed down upon LAB-CFCS application under infection suggests that LAB-CFCS up-regulated defense-related secondary metabolism, reinforcing structural barriers and phenolic-based antimicrobial defenses with minimal activity of both enzymes. These results are consistent with earlier reports that beneficial microbes can act as biological elicitors, priming the host’s defense system against pathogens [78]. Thus, the synergistic effects between LAB and plant antioxidant machinery suggest that LAB enhance systemic resistance by balancing ROS production and scavenging, thereby reducing disease severity [93].

Biochemically, the plant undergoes several modifications in response to the bacterial invasion.

Phenylalanine ammonia-lyase is a key enzyme in the phenylpropanoid pathway and plays an important role deamination of L-phenylalanine to trans-cinnamic acid, which serves as the primary precursor for a wide range of secondary metabolites, including lignin, flavonoids, tannins, anthocyanins, terpenoids and various phenolics. These metabolites are crucial for both structural reinforcement and defense mechanisms in plants. In the present study, infected plants treated exhibited elevated PAL activity compared with untreated healthy plants; however, the levels of phenolics, flavonoids, and anthocyanins remained lower than those observed in the corresponding healthy treated plants. In this regard, the phenolics-oxidizing enzyme, PPO, recorded the highest stimulation under pathogen stress [91]. Thus, any increment of PAL down steam metabolites could be oxidized by PPO. This observation suggests that although PAL activity was stimulated under pathogen stress, the synthesized metabolites may have been rapidly utilized or redirected toward defense-related processes, including lignification, reinforcement of cell walls, and detoxification responses by PPO against *R. solanacearum* infection [94]. This observation recommended under LAB-CFCS where the activity of PPO reduced especially under LS and LR, but the activity of PAL is still higher than healthy plants along with recording the highest levels of secondary metabolites. The increase in PAL activity, with low level of PPO, suggests an enhanced flux into the phenylpropanoid pathway, which likely contributes to the biosynthesis of lignin and phenolic compounds [95]. The increases in PAL activity (1.3–1.7 fold) across LAB-CFCS treatment to infected plants further indicate that even subtle stimulation of this pathway can contribute to a more robust defense system, including the synthesis of phytoalexins, which are directly involved in pathogen resistance [94]. These compounds provides potent antimicrobial and antioxidant activities, helping the plant mitigate oxidative damage caused by biotic stress. Overall, the balance between PAL and PPO activities reflects an active defense strategy, reinforcing both structural integrity through chemical defenses via phenolics and flavonoids [96, 97]. The plant defense responses are complex and the observed protection in LAB-treated plants is likely associated with multiple coordinated defense mechanisms, including enhanced antioxidant capacity, modulation of signaling molecules, maintenance of membrane integrity, restriction of pathogen-induced oxidative damage and balance of PAL and PPO activities.

Taken together, these findings reveal a coordinated regulatory role of LAB in plant defense signaling. By restoring SA and H₂S, chlorophyll, moderating NO and ROS accumulation, and enhancing antioxidants, and secondary metabolites, LAB rebalance the defense network disrupted by *R. solanacearum*. This not only strengthens direct pathogen resistance but also improves metabolic resilience, allowing plants to maintain redox balance and effective immune signaling under pathogen challenge. Future work should focus on unraveling the underlying defense- and hormone-related gene expression using RT-PCR would provide deeper molecular insight into the mechanisms underlying LAB-CFCS-mediated resistance against *Ralstonia solanacearum*.

## Conclusion

The present study demonstrated that LAB-CFCS treatments effectively inhibited both the growth and biofilm formation of *R. solanacearum in vitro*. The LAB-CFCS also alleviated the detrimental effects of *R. solanacearum* on photosynthetic pigments and organic acid metabolism while simultaneously enhancing the fundamental physiological functions of infected plants. The application of LAB-CFCS alleviated ROS accumulation and lipid peroxidation, highlighting their potential as biocontrol agents that maintain redox homeostasis and enhance host resistance through both activation of antioxidative pathways and upregulation of secondary metabolism. The synergistic restoration of SA and H₂S highlights the multifaceted role of LAB in strengthening both hormonal and gasotransmitter-mediated defenses. LAB-CFCS alleviated pathogen pressure via reprogrammed metabolic responses, leading to higher accumulation of protective compounds. This dual role as preventive in healthy plants and curative under infection, highlights LAB as promising eco-friendly agents for enhancing plant resilience.

## Data Availability

The authors declare that the data supporting the findings of this study are available within the paper and its Supplementary Information. Further information is available from the corresponding author upon reasonable request.
